# Implications of Wastewater Surveillance for Clinical Care among Infectious Disease Physicians, United States, 2024

**DOI:** 10.3201/eid3213.260203

**Published:** 2026-08

**Authors:** Shantrice L. Jones, Angela D. Blackwell, Carly Adams, Philip M. Polgreen, Susan E. Beekmann, Chelsea Gridley-Smith, Jessica N. Ricaldi, Scott Santibañez

**Affiliations:** Centers for Disease Control and Prevention, Atlanta, Georgia, USA (S.L. Jones, A.D. Blackwell, C. Adams, J.N. Ricaldi, S. Santibañez); The University of Iowa Roy J. and Lucille A. Carver College of Medicine, Iowa City, Iowa, USA (P.M. Polgreen, S.E. Beekmann); National Association of County and City Health Officials, Washington, DC, USA (C. Gridley-Smith)

**Keywords:** wastewater surveillance, clinical care, infectious disease, United States

## Abstract

We analyzed 192 responses from a 2024 survey of Emerging Infections Network members in the United States regarding how wastewater surveillance can affect clinical practice. Four themes emerged: situational awareness, patient counseling, infection control, and diagnostic support. Enhanced collaboration between public health officials and clinicians might optimize utility of such surveillance in clinical practice.

Although wastewater surveillance (WS) (*1*) has a role in outbreak detection (*2*–*4*), integration of WS data into clinical practice remains limited (*5*). Little is known about how infectious disease clinicians perceive WS (*6*). The Centers for Disease Control and Prevention (CDC) and the Infectious Diseases Society of America’s Emerging Infections Network (EIN) (https://ein.idsociety.org), a sentinel network of infectious disease physicians and other infectious disease specialists, surveyed EIN member-clinicians in 2024 to generate hypotheses about knowledge of, attitudes toward, and use of WS data (*5*,*7*) Our previously published quantitative results revealed 56% of EIN clinicians did not review WS data regularly, 22% of respondents reviewed it regularly, and 22% were not aware of WS data (*5*). To complement those previous findings, we present an in-depth analysis of qualitative data from the same survey.

## Methods

During February–March 2024, we distributed a 9-question, cross-sectional, voluntary internet survey to EIN members. We followed applicable reporting guidelines from The Checklist for Reporting Results of Internet E-Surveys and Standards for Reporting Qualitative Research (*8*). We focused on this open-ended question: “Please provide any specific example(s) of how wastewater surveillance has affected/could affect your clinical practice.” Of 1,809 EIN members, 448 (25%) responded to the survey. Of those who responded, 192 (40%) provided free-text comments about clinical impact, with 178 describing how WS does or could affect their clinical practice and 14 indicating WS has no affect on their clinical practice. Using content analysis, analysts iteratively reviewed the 192 responses using an inductive approach, noting multiple response topics. Analysts then coded responses, identified potential themes that aligned with responses, and selected representative quotations from a password-protected file. We reviewed codes and themes and refined them through team discussions to ensure consistency in interpretation. The CDC’s National Center for Emerging and Zoonotic Infectious Diseases Human Subjects Advisor reviewed this activity and deemed it nonresearch per applicable federal law and CDC policy.

## Results

We compiled key themes based on survey responses (Figure). Of 192 responses, approximately half (47%, n = 91) involved situational awareness and early warning. Respondents stated WS can help to “identify outbreaks earlier,” “act like a ‘canary in a coal mine’ as far as being a harbinger of diseases in a community,” and can help to “notify emergency department physicians to be on alert for certain infections.” About one quarter (24%, n = 47) of responses pertained to infection prevention and control. One respondent reported using WS “with hospital planning as part of my role as Medical Director for Infection Control in my hospital system.” A hospital epidemiologist noted using WS “to help gauge need for sending systemwide updates and reminders about isolation, diagnostic tools, immunizations, etc.”

**Figure:**
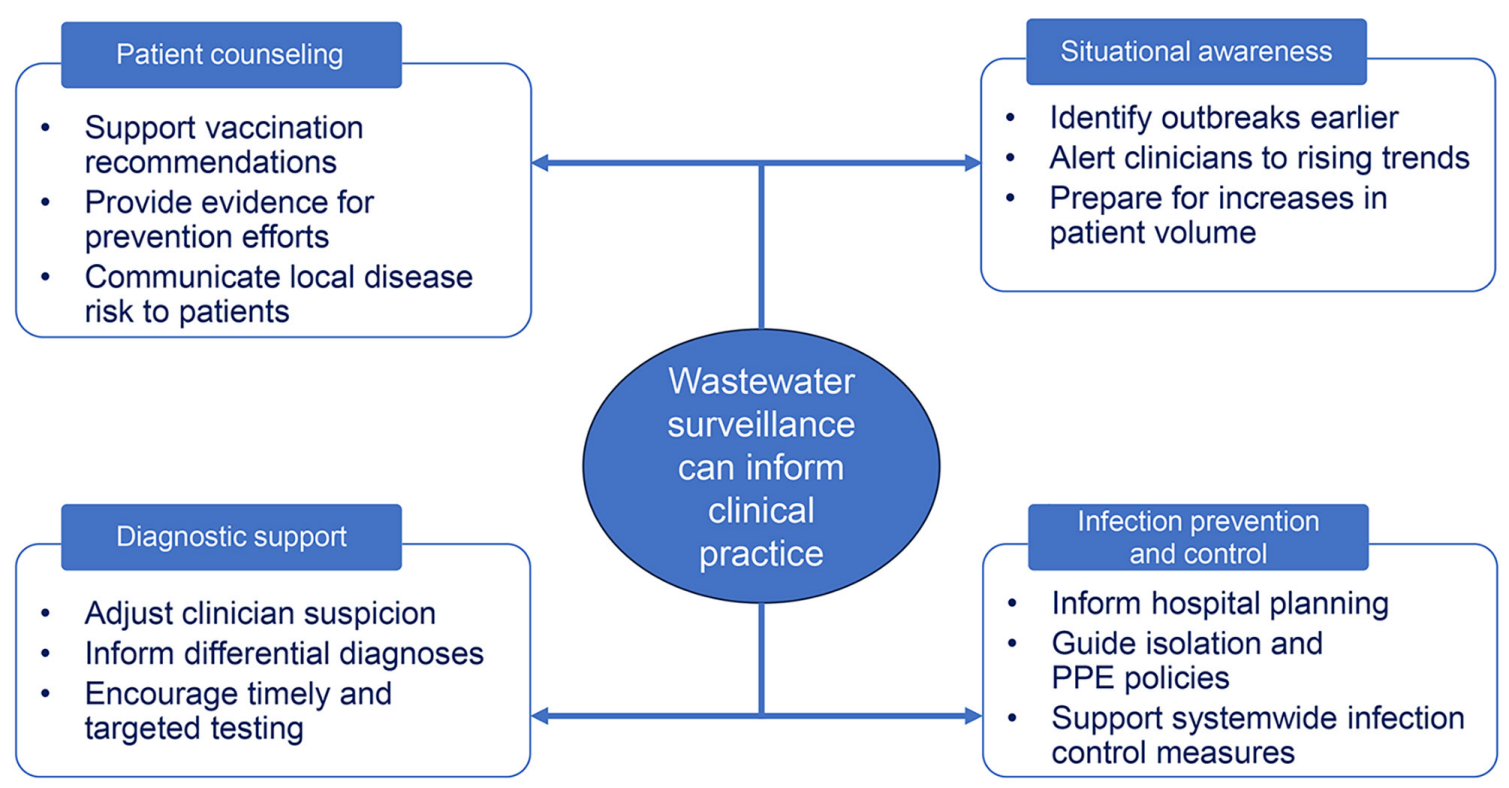
Wastewater surveillance information utilization inferred from an investigation into the implications of wastewater surveillance for clinical care among infectious disease physicians, United States, 2024. PPE, personal protective equipment.

Fifteen percent (n = 29) of responses noted the importance of WS in diagnostic testing and differential diagnoses. Respondents described how awareness “raises or lowers my clinical suspicion” and “can encourage faster relevant testing.” Thirteen percent (n = 24) of respondents described the value of WS in educating patients. Specifically, respondents described how WS can provide “evidence for encouraging prevention, including vaccination,” can be “helpful [in being] able to cite rising rates when recommending vaccination,” and can be “very helpful in encouraging immunizations in those who don’t believe a pathogen is a local issue.” Last, respondents described how awareness of antibiotic resistance “may lower my threshold to use broader empiric antibiotics” and may aid in “management of vulnerable patient populations, such as transplant recipients and other immunocompromised [patients].”

A minority of respondents expressed uncertainty about WS clinical utility or noted that usefulness depends on specific conditions. For example, some respondents noted that its value depends on timeliness, geographic specificity, and clear clinical guidance. Five respondents indicated that more guidance is needed from health departments. One clinician wrote, “mainly, wastewater surveillance should be [managed by the] health department, with rapid dissemination of info to [infectious disease] docs,” and another said WS could provide “data the state health department could use in some manner to provide guidance to physicians and the public.”

## Discussion

In this analysis, most respondents identified positive elements of how WS can be used. During the analysis and review process, we noted the phrasing of the question did not make a clear distinction between respondents’ impressions of how WS findings are actively incorporated into their clinical practice versus their perceptions of how WS can be useful. Although perceived potential usefulness is still valuable, a limitation of our analysis is that findings should not be interpreted as evidence that WS has been broadly adopted. This limitation is supported by the frequent use of conditional language by respondents (e.g., “could,” “would,” “may”), suggesting that many comments reflected perceived potential rather than current routine use. When considered together with our previous findings from the same survey (*5*), those comments may help identify hypotheses about potential early uses of WS in select clinical or health-system settings. The brief free-text responses limited the depth of insight. A follow-up study using in-depth interviews or focus groups could help more clearly delineate these aspects. By design, the methods of analysis we employed were intended for hypothesis generation and do not allow the drawing of conclusions. Also, respondents with greater interest in WS may have been more likely to provide comments. Overall, we believe that our findings suggest the potential of WS to inform many aspects of clinical practice.
